# External Model Performance Evaluation of Twelve Infliximab Population Pharmacokinetic Models in Patients with Inflammatory Bowel Disease

**DOI:** 10.3390/pharmaceutics13091368

**Published:** 2021-08-31

**Authors:** Christina Schräpel, Lukas Kovar, Dominik Selzer, Ute Hofmann, Florian Tran, Walter Reinisch, Matthias Schwab, Thorsten Lehr

**Affiliations:** 1 Clinical Pharmacy, Saarland University, 66123 Saarbrücken, Germany; christina.schraepel@uni-saarland.de (C.S.); lukas.kovar@uni-saarland.de (L.K.); dominik.selzer@uni-saarland.de (D.S.); 2 Dr. Margarete Fischer-Bosch-Institute of Clinical Pharmacology, University of Tübingen, 70376 Stuttgart, Germany; ute.hofmann@ikp-stuttgart.de (U.H.); matthias.schwab@ikp-stuttgart.de (M.S.); 3 Institute of Clinical Molecular Biology, Kiel University and University Medical Center Schleswig-Holstein, 24105 Kiel, Germany; f.tran@ikmb.uni-kiel.de; 4 Department of Internal Medicine I, University Medical Center Schleswig-Holstein, 24105 Kiel, Germany; 5 Department of Internal Medicine III, Medical University of Vienna, 1090 Vienna, Austria; walter.reinisch@meduniwien.ac.at; 6 Departments of Clinical Pharmacology, Pharmacy and Biochemistry, University of Tübingen, 72076 Tübingen, Germany

**Keywords:** infliximab, population pharmacokinetics, inflammatory bowel disease, model-informed precision dosing, dose individualization

## Abstract

Infliximab is approved for treatment of various chronic inflammatory diseases including inflammatory bowel disease (IBD). However, high variability in infliximab trough levels has been associated with diverse response rates. Model-informed precision dosing (MIPD) with population pharmacokinetic models could help to individualize infliximab dosing regimens and improve therapy. The aim of this study was to evaluate the predictive performance of published infliximab population pharmacokinetic models for IBD patients with an external data set. The data set consisted of 105 IBD patients with 336 infliximab concentrations. Literature review identified 12 published models eligible for external evaluation. Model performance was evaluated with goodness-of-fit plots, prediction- and variability-corrected visual predictive checks (pvcVPCs) and quantitative measures. For anti-drug antibody (ADA)-negative patients, model accuracy decreased for predictions > 6 months, while bias did not increase. In general, predictions for patients developing ADA were less accurate for all models investigated. Two models with the highest classification accuracy identified necessary dose escalations (for trough concentrations < 5 µg/mL) in 88% of cases. In summary, population pharmacokinetic modeling can be used to individualize infliximab dosing and thereby help to prevent infliximab trough concentrations dropping below the target trough concentration. However, predictions of infliximab concentrations for patients developing ADA remain challenging.

## 1. Introduction

Infliximab is an intravenously administered recombinant chimeric monoclonal antibody that inhibits both soluble and membrane-bound tumor necrosis factor alpha (TNF-α) [1]. Infliximab is approved for treatment of various chronic inflammatory diseases including the inflammatory bowel diseases (IBD) Crohn’s disease (CD) and ulcerative colitis (UC) [2,3]. After its approval in 1999 by the European Medicines Agency (EMA), infliximab revolutionized the treatment of CD and UC because of its ability to induce long-term remission, reduce hospitalizations, and restore quality of life [4,5]. Today, infliximab is still widely used and available as different biosimilars [3].

Infliximab exhibits linear pharmacokinetic behavior, while low trough concentrations are associated with impaired or even loss of response to infliximab therapy [6,7,8,9]. UC patients with detectable serum infliximab trough concentrations showed a 4 times higher probability and CD patients even a 13 times higher probability of being in clinical remission, making serum infliximab levels a predictor of clinical response [6,9,10,11,12]. According to a recent guideline from the American Gastroenterological Association Institute, infliximab should be dosed to achieve target trough concentrations of ≥ 5 µg/mL in order to improve therapy outcome [13]. However, a high inter-individual variability in infliximab trough levels has been observed contributing to a high rate of treatment failure [9,12,13,14]. About 10–40% of patients fail to respond to induction therapy (primary non-response) [15], and subsequently, 13% of patients lose response annually after initially responding (secondary non-response) [16]. One of the reasons for primary and secondary non-response is the formation of anti-drug antibodies (ADAs) against infliximab, leading to an increased infliximab clearance (CL) [9,17,18].

A good understanding of the high variability in infliximab trough levels is essential for dose individualization strategies [4,19,20,21,22]. In the past, several efforts have been made to characterize infliximab pharmacokinetics (PK), including the quantification and explanation of inter-individual variability, and to develop population pharmacokinetic models for dose individualization [21,23,24,25,26,27]. While these analyses identified various covariates (e.g., albumin levels, sex, weight, ADA development, and use of concomitant immunomodulators) that influence infliximab CL and volume of distribution (V_d_), the covariates could only partly explain the observed inter-individual and inter-occasion variability (IOV) [23,24,26,27].

Thus, population pharmacokinetic models combined with data from therapeutic drug monitoring could help to optimize drug dosing regimens in individual patients via model-informed precision dosing (MIPD) [28,29,30,31,32]. Infliximab models have recently been used to simulate dosing regimens for different patient populations or for evaluation of individualized dose adjustments and incidences of loss of response [31,33,34]. However, a comprehensive external evaluation of the different infliximab population pharmacokinetic modeling approaches including assessment of accuracy and bias of model predictions over time as well as the ability to predict the need for dose escalation is still pending. Hence, the aim of this work was to provide an overview of published infliximab population pharmacokinetic models for patients with IBD as well as to evaluate and compare model performance with a focus on differences between ADA-negative and ADA-positive subpopulations in a Bayesian forecasting setting using an external data set.

## 2. Materials and Methods

### 2.1. External Evaluation Data Set

For predictive external model evaluation, data originated from a previously published observational study that was reviewed and approved by the institutional review board of the Medical University of Vienna [35]. All participating patients had signed an informed consent form.

Patients with an established diagnosis of CD and UC were enrolled in the study. All participants had previously responded to induction therapy, receiving three infusions at weeks zero, two, and six, and were assigned to a maintenance dosing regimen. Serum samples of patients (median 2, range 1–12 samples) were collected during infliximab therapy at both midpoint and trough times of a dosing interval while exact time points were not specified in the protocol. Laboratory and demographic data were collected, including serum infliximab concentrations, ADA levels, serum albumin concentrations, C-reactive protein levels, weight, use of concomitant immunomodulators, Harvey–Bradshaw index (HBI), and smoking status.

Serum infliximab concentrations used in this analysis were measured with a commercially available enzyme-linked immunosorbent assay (ELISA) method (Immundiagnostik Germany, Bensheim, Germany) with a lower limit of quantification (LLOQ) of 2.68 ng/mL [36]. ADA concentrations were determined using the homogeneous mobility shift assay (HMSA) from Prometheus Labs Inc., San Diego, CA, USA with an LLOQ of 3.13 U/mL [37]. Patients were assigned to the ADA-positive patient cohort if any measured ADA concentration was above the threshold of 6.6 U/mL [37,38].

### 2.2. Population Pharmacokinetic Models and Software

A comprehensive and systematic literature search in PubMed was performed for infliximab population pharmacokinetic analyses in patients with IBD. The search terms were “infliximab” AND “population” AND “pharmacokinetics” and reference lists of identified articles were manually screened for further eligible studies. Subsequently, modeling and study information was collected, including model structure, population pharmacokinetic parameter values, covariates, inter-individual variability, residual variability, information on patient cohorts, disease type, number of patients, number of collected blood samples, and ADA immunogenicity rate. The population pharmacokinetic models described in the gathered studies were implemented and evaluated using the nonlinear mixed effects modeling software NONMEM^®^ version 7.4 (Icon Development Solutions, Ellicott City, MD, USA). Computations for prediction- and variability-corrected visual predictive checks (pvcVPCs) were generated with the PsN (version 4.9.0) tool “vpc” [39,40]. Data management, graphics, and quantitative model diagnostics were carried out using the R programming language version 3.6.3 (R Foundation for Statistical Computing, Vienna, Austria) and R Studio^®^ version 1.2.5019 (R Studio, Inc., Boston, MA, USA).

### 2.3. Model Performance Evaluation

For the implemented population pharmacokinetic models, all parameters (fixed and random effects) were set to published values of the respective study. To assess the potential applications in a clinical setting, model performances to predict serum infliximab concentrations with a Bayesian approach were evaluated with the external data set. Here, the first measured serum infliximab concentration of each patient (C_MAP_) was used for maximum a posteriori (MAP) estimation of individual pharmacokinetic parameters (empirical Bayes pharmacokinetic parameter estimates [EBEs]) considering interaction between inter-individual variability and residual variability for prediction of subsequent serum infliximab concentrations (Bayesian forecasting). As recommended by Abrantes and coworkers, IOV was included in the estimation of EBEs but excluded in the calculation of individual pharmacokinetic parameters used for predictions [41]. For prospective predictions, individual patient covariates for times after C_MAP_ were imputed using last observation carried forward.

Visual and quantitative methods were applied for the evaluation of predictive model performances. Goodness-of-fit plots of individual predicted infliximab concentrations vs. observed infliximab concentrations were generated for visual evaluation. Moreover, two quantitative measures were calculated, including the median symmetric accuracy (ζ, Equation (1)) and the symmetric signed percentage bias (SSPB, Equation (2)) to evaluate the model regarding prediction accuracy and prediction bias.

(1) $$\zeta =100\times \left[e^{\left(\mathrm{median}\left(\left|\ln\left(\frac{y_{i}}{x_{i}}\right)\right|\right)\right)}-1\right],$$

(2) $$\mathrm{SSPB}=100\times \left[\mathrm{sign}\left(\mathrm{median}\left(\ln\left(\frac{y_{i}}{x_{i}}\right)\right)\right)\right]\times \left[e^{\left(\left|\mathrm{median}\left(\ln\left(\frac{y_{i}}{x_{i}}\right)\right)\right|\right)}-1\right].$$

In Equations (1) and (2) *x_i_* represents the *i*th observed infliximab serum concentration and *y_i_* the corresponding predicted serum concentration.

ζ represents the typical absolute percentage error with 50% of absolute percentage errors below *ζ* [42]. The SSPB, a measure of bias, estimates the central tendency of the error penalizing underprediction and overprediction equally as illustrated by Morley and coworkers [42].

As mentioned before, dose escalation can be beneficial in patients with trough concentrations below the target threshold of 5 µg/mL. Hence, a model’s ability to correctly predict the need for dose escalation was further investigated. For that, observed and predicted trough concentrations were split into two categories: C_trough_ < 5 µg/mL (dose escalation needed) and C_trough_ ≥ 5 µg/mL (no dose escalation needed). Correct predictions of need for dose escalation are referred to as “true positive” while correct predictions of no need for dose escalation are referred to as “true negative”. Model accuracy, i.e., the fraction of observed and corresponding predicted trough concentrations, both <5 µg/mL or both ≥ 5 µg/mL, were calculated for all models. Here, model classification performance was evaluated for trough samples in which ADA status was negative and for trough samples in which ADA status was positive individually.

In addition, pvcVPCs were performed with multiple replicates (n = 1000) of the study population. The simulated concentrations (median, 5th, and 95th percentiles), the corresponding 95% confidence intervals as well as prediction- and variability-corrected observed concentrations (with median, 5th, and 95th percentiles) were plotted against time after dose.

## 3. Results

### 3.1. Characteristics of Published Population Pharmacokinetic Models of Infliximab in Patients with IBD

The comprehensive literature search in PubMed for population pharmacokinetic analyses of infliximab in patients with IBD revealed 25 population pharmacokinetic models, which are listed in Table 1 together with the respective model characteristics. The models partially differ both in base model structure as well as tested and integrated covariates. The majority of the studies used a 2-compartment model (n = 18) with first-order elimination, while seven models implemented a 1-compartment model. Yet, five out of seven studies that used a 1-compartment base model were developed with sparse data including only infliximab trough samples in the model building process.

Integrated covariates on infliximab CL and central volume of distribution (V_c_) include patient characteristics (sex, weight, and age), clinical characteristics (albumin levels, HBI, ADA status, etc.) as well as concomitant medication of immunomodulators (IMM).

Of the 25 models, 14 included albumin concentrations, 14 weight, and four sex as a covariate on CL. Moreover, four models included an IOV for the CL parameter. Eighteen models integrated ADA as a covariate (sixteen as binary, one as ordinal, and one as continuous covariate), two models implemented a risk function of developing ADA, and three did not include ADA status in the model since only a small fraction of patients in the respective model building data set were ADA positive (≤3%). Two studies did not include ADA-positive patients for the model building process (see Table 1).

Furthermore, model building data sets vastly differed in patient and sample numbers, patient cohort (patients with CD/UC; adult/pediatric patients) as well as sampling times (see Table 1). Eleven models used data from both patients with CD and UC, eleven from patients with CD, and three from patients with UC. The majority of models were developed with data from adult patients (19/25), three with data from both adult and pediatric patients, and three with data from pediatric patients only.

### 3.2. Eligible Population Pharmacokinetic Models for Evaluation

Twelve out of 25 population pharmacokinetic models (entries marked with an asterisk in Table 1) were eligible for model performance evaluation with the external data set. From Fasanmade et al., 2011, two of three models that were developed using a data set of adult patients and a data set of both pediatric and adult patients, respectively, were included in the analysis [24]. Edlund et al. published three different approaches for handling the ADA covariate [43]. All three models based on ADA measurements by HMSA were included in the analysis and are, hereafter, referred to as I (ADA covariate on the patient level), II (ADA covariate on sample level), and III (ADA concentrations as a continuous covariate). Eleven out of 25 models (Ternant et al., 2008 [44], Dotan et al., 2014 [45], Ternant et al., 2015 [46], Brandse et al., 2017 [47], Kevans et al., 2018 [48], Dreesen et al., 2019 [49], Matsuoka et al., 2019 [50], Petitcollin et al., 2019 [51], Dreesen et al., 2020 [27], Bauman et al., 2020 [21], and Kantasiripitak et al., 2021 [26]) could not be evaluated because of data set incompatibility (e.g., missing covariates in our data set) or lack of reported model implementation details. The model by Grišić et al. was not included in the analysis as it was specifically focused on modeling the effects of pregnancy affecting infliximab PK [52]. In summary, models developed by Aubourg et al., 2015 [53], Buurman et al., 2015 [54], Brandse et al., 2016 [55], Edlund et al., 2017 (I–III) [43], Fasanmade et al., 2009 [23], Fasanmade et al., 2011 (adults and adults/children) [24], Passot et al., 2016 [56], Petitcollin et al., 2018 [25], and Xu et al., 2012 [57] were implemented and included in the external evaluation. Additional information on the investigated models regarding assumptions for model implementation (e.g., handling of missing units or ambiguities) are outlined in Section 1 of the Supplementary Materials.

### 3.3. External Evaluation Data Set

Four hundred serum infliximab concentrations from 124 patients were available in the data set. Data from 11 patients (33 infliximab concentrations total) were excluded because of insufficient information on the respective dosing regimen (e.g., unknown time of dosing). Three concentrations below the LLOQ (<1% of samples) were excluded from the external evaluation (M1 method) [58]. Moreover, 28 concentrations classified as pharmacokinetically implausible (concentrations that did not decrease over a sampling period of at least seven days within a dosing interval) were removed from the analysis. Consequently, eight patients lacked informative infliximab PK data, i.e., at least one sample with detectable infliximab concentrations, and were therefore excluded from the analysis.

As a result, a total of 336 infliximab concentrations from 105 patients with IBD, including 76 cases of CD and 29 cases of UC, were available for external evaluation (median number of infliximab samples per patient: 2; range: 1–12). Twenty-two patients had at least one positive ADA sample. In total, ADA levels above the threshold of 6.6 U/mL were measured in 49 samples. An overview of clinical and demographic patient characteristics of the external data set is presented in Table 2. Infliximab was administered to patients using various dosing regimens with a median (interquartile range (IQR)) infliximab dose of 5.5 (5.1–5.9) mg/kg and a median (IQR) dosing interval of 8.0 (7.7–8.6) weeks.

**Table 1:** Overview of published pharmacokinetic models for infliximab in patients with IBD.

Publication	CD/UC	Patient Cohort	No. of Patients (Samples)	Sampling Times	Base Model	Covariates on CL	Covariates on V_c_	IOV	Induction/ Maintenance ^1^	Inclusion of ADA+ Patients	Ref.
Ternant et al., 2008	both	adults	33 (478)	peak, trough	2-CMT	ADA	sex, weight	-	both	yes (15%)	[44]
Fasanmade et al., 2009 *	UC	adults	482 (4145)	peak, midpoint, trough	2-CMT	ADA, alb, sex	sex, weight	-	both	yes (7%)	[23]
Fasanmade et al., 2011 (a) *	CD	adults	580 (/)	peak, midpoint, trough	2-CMT	ADA, alb, IMM, weight	weight ^2^	CL	both	yes (11%)	[24]
Fasanmade et al., 2011 (c)	CD	children	112 (/)	peak, midpoint, trough	2-CMT	alb, weight	weight ^2^	CL	both	yes (3%)	[24]
Fasanmade et al., 2011(a/c) *	CD	both	692 (5757)	peak, midpoint, trough	2-CMT	ADA, alb, IMM, weight	weight ^2^	CL	both	yes (10%)	[24]
Xu et al., 2012 *	both	both	655 ^3^ (/)	/	2-CMT	ADA, alb, weight ^4^	weight ^2^	-	/	yes (/)	[57]
Dotan et al., 2014	both	adults	54 (169)	trough	2-CMT	ADA, alb, weight ^4^	weight ^2^	-	both	yes (31%)	[45]
Aubourg et al., 2015 *	CD	adults	133 (/)	trough, peak	2-CMT	sex	sex, weight	-	treatment initiation	no	[53]
Buurman et al., 2015 *	both	adults	42 (188)	trough	2-CMT	ADA, period ^5^, sex	HBI	-	both	yes (5%)	[54]
Ternant et al., 2015	CD	adults	111 (546)	throughout dosing interval	1-CMT	FCGR3A-158V/V, hsCRP	-	-	maintenance	yes (2%)	[46]
Brandse et al., 2016 *	UC	adults	19 (/)	throughout dosing interval	2-CMT	ADA, alb	-	-	induction	yes (32%)	[55]
Passot et al., 2016 *	both	both	79 ^6^ (/)	trough	1-CMT	CD/UC, sex, weight	CD/UC, sex, weight	-	both	no	[56]
Brandse et al., 2017	both	adults	332 (997)	throughout dosing interval	2-CMT	ADA, alb, previous exposure, weight ^4^	weight ^2^	-	both	yes (23%)	[47]
Edlund et al., 2017(I–III) *^,7^	CD	adults	68 (152)	midpoint, trough	2-CMT	ADA ^8^, weight ^4,9^	weight ^2,9^	-	maintenance	yes (37%)	[43]
Kevans et al., 2018	both	adults	51 (/)	throughout dosing interval	2-CMT	ADA, alb, weight ^4^, time-varying CL ^10^	weight ^2^	-	induction	yes (11%)	[48]
Petitcollin et al., 2018 *	CD	children	20 (145)	trough	1-CMT	alb, time-varying CL/risk of immunization ^11^	-	-	both	yes (15%)	[25]
Dreesen et al., 2019	UC	adults	204 (583)	trough	1-CMT	alb, CRP, Mayo	FFM, CS, panc.	CL	induction	yes (1%) ^12^	[49]
Matsuoka et al., 2019	CD	adults	121 (832)	trough	1-CMT	ADA, alb, weight	-	-	maintenance	yes (26%)	[50]
Petitcollin et al., 2019	both	adults	91 (607)	trough	1-CMT	CD/UC, CRP, dose, Mayo, AZA, time-varying CL/risk of immunization ^11^, weight ^13^	-	-	maintenance	yes (1%)	[51]
Bauman et al., 2020	both	children	135 (289)	trough	2-CMT	ADA ^14^, alb, ESR, weight	weight ^2^	-	maintenance	yes (62%)	[21]
Dreesen et al., 2020	CD	adults	116 (1329)	midpoint, trough	2-CMT	ADA, alb, CDAI, fCal	-	-	both	yes (18%)	[27]
Grišić et al., 2020	both	pregnant	19 (172)	throughout dosing interval	1-CMT	ADA, 2nd/3rd trimester	-	-	both	yes (30%) ^12,15^	[52]
Kantasiripitak et al., 2021	both	adults	104 (272)	trough	2-CMT	ADA, age, alb, CRP, FFM	-	-	induction	yes (13%)	[26]

Note: -: none; /: unknown; (a): (adults); (a/c): (adults/children); ADA: anti-drug antibodies; ADA+: anti-drug antibody positive; alb: albumin concentrations; AZA: azathioprine; (c): children; CD: Crohn’s disease; CDAI: Crohn’s disease activity index; CL: clearance; CMT: compartment; CRP: C-reactive protein; CS: corticosteroids; ESR: erythrocyte sedimentation rate; fCal: fecal calprotectin; FCGR3A-158V/V: Fc fragment of IgG, low affinity IIIa, receptor (CD16a) polymorphism; FFM: fat-free mass; HBI: Harvey–Bradshaw index; hsCRP: high-sensitivity C-reactive protein; IBD: inflammatory bowel disease; IMM: immunomodulators; IOV: inter-occasion variability; Mayo: Mayo score; No.: number; panc.: pancolitis; Ref.: reference; UC: ulcerative colitis; V_c_: volume of central compartment; * included in the external model performance evaluation; ^1^ blood sample data collected during induction and/or maintenance therapy; ^2^ covariate also on volume of peripheral compartment (V_p_); ^3^ 133 more pediatric patients with other inflammatory diseases were included; ^4^ covariate also on intercompartmental clearance (Q); ^5^ induction or maintenance phase; ^6^ 139 more patients with other inflammatory diseases were included; ^7^ three similar models with different handling of the ADA covariate; ^8^ ADA as binary or continuous covariate; ^9^ allometric scaling; ^10^ a component of CL that varies over time independent of patient factors; ^11^ describing varying infliximab CL over time (independent from ADA testing); ^12^ percentage of ADA-positive blood samples; ^13^ as a covariate on the CL increase over time; ^14^ ADA was included as an ordinal covariate with four categories; ^15^ samples with infliximab concentrations ≤5 µg/mL were assessed for ADAs.

### 3.4. Predictive Model Evaluation Goodness-of-Fit Plots

The first concentration (C_MAP_) was used for MAP estimation of EBEs, and all subsequent concentrations were predicted. Data was split into two sets of ADA-positive and ADA-negative patients. Additionally, for ADA-negative patients, predictions were stratified for different time intervals after C_MAP_ (i.e., “within 1 month”, “between 1 and 6 months” and “>6 months”). For ADA-positive patients, predicted concentrations were stratified as follows: infliximab concentrations for patients that have not been tested ADA positive yet (“before ADA+”), concentrations measured within one month or at first ADA detection (“1st time ADA+ and ≤1 month”), and concentrations measured after one month of first ADA detection (“>1 month of being ADA+”).

**Figure 1:**
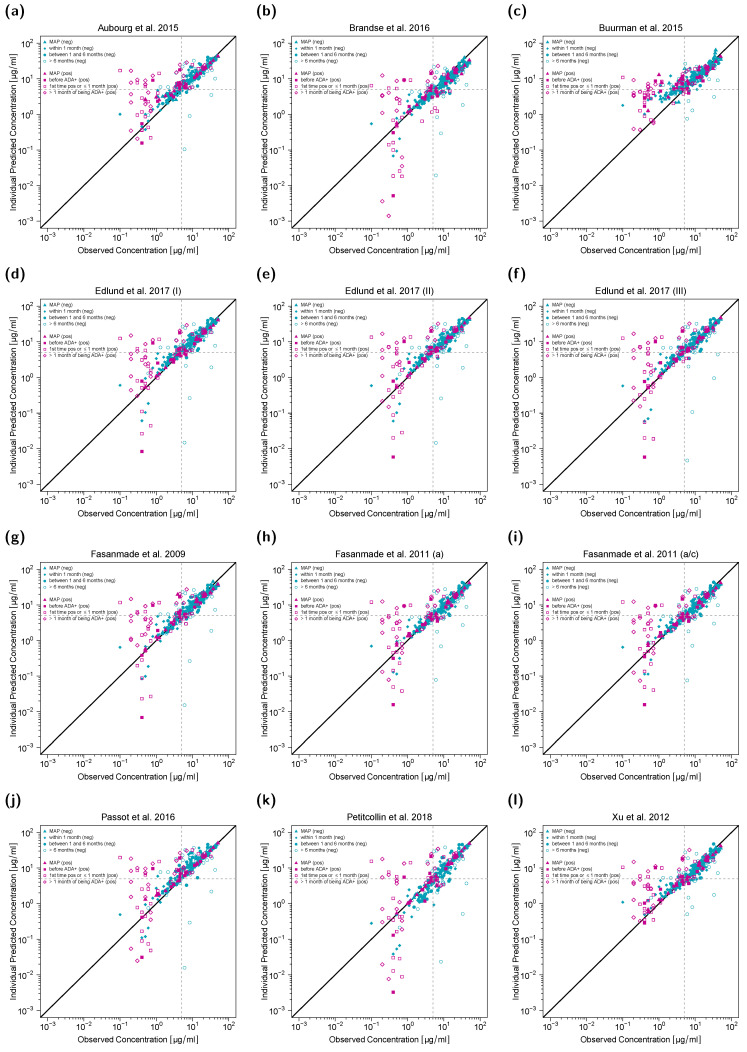
Individual predicted versus observed serum infliximab concentrations for twelve different population pharmacokinetic models (**a**–**l**). Concentrations of anti-drug antibody (ADA) negative patients are shown in turquoise, concentrations of ADA-positive patients in pink. Concentrations used for maximum a posteriori (MAP) estimation (C_MAP_) are depicted as triangles, the remaining symbols depict predictions in different time intervals after C_MAP_. Black solid lines represent the lines of identity, gray dashed lines mark the target trough concentration of 5 µg/mL. In (**k**), one serum concentration falls outside the plotting range but is included in the full plot depicted in the Supplementary Materials. a: adults; a/c: adults/children; (neg): ADA-negative patients; (pos): ADA-positive patients.

Goodness-of-fit plots (Figure 1) show that model predictions of infliximab concentrations for most ADA-negative patients (turquoise symbols) matched precisely with the observed concentrations. However, predictions of concentrations of ADA-positive patients (pink symbols), especially those measured within and after one month of first ADA detection, were less accurate (turquoise symbols). Additionally, in ADA-negative patients, predictions of concentrations measured more than six months after C_MAP_ showed larger deviation from the corresponding observed concentrations compared to predictions of concentrations measured within the first six months after C_MAP_ in this study setting.

### 3.5. Accuracy and Bias of Model Predictions

**Figure 2:**
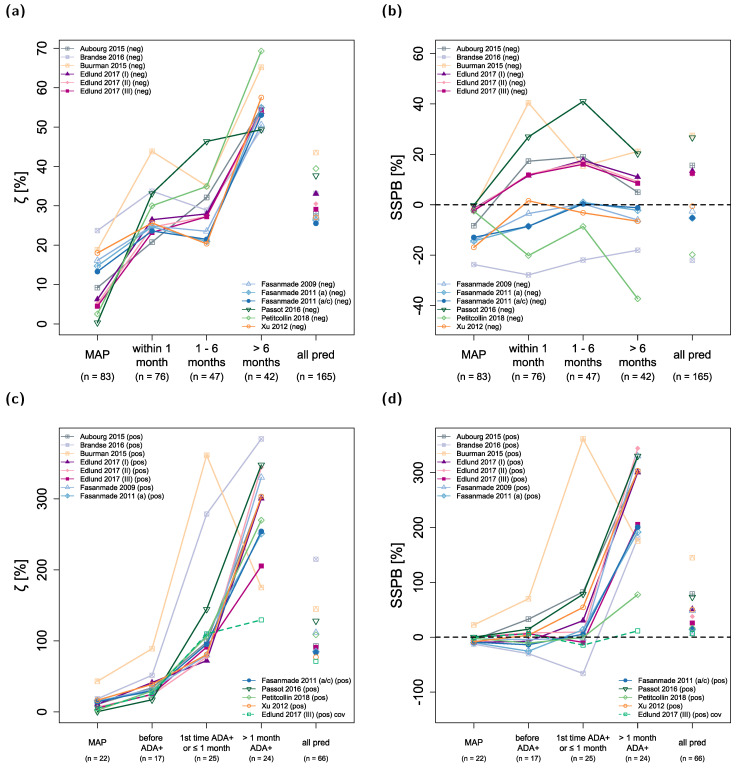
Model prediction accuracy (ζ, (**a**,**c**)) and bias (SSPB, (**b**,**d**)) over time. The upper panel shows results for anti-drug antibody (ADA) negative patients, the lower panel for ADA-positive patients. Numbers in parentheses refer to the number of observed concentrations in the respective time interval. “all pred” covers all predicted concentrations excluding concentrations used for maximum a posteriori (MAP) estimation (C_MAP_) of individual pharmacokinetic parameters. Solid lines depict the results for model predictions using patient covariates determined at the time of C_MAP_. The green dashed line shows the results for predictions with the model by Edlund et al., 2017 (III), using measured time-varying covariates. a: adults; a/c: adults/children; ADA+: anti-drug antibody positive; cov: covariates; (neg): ADA-negative patients, (pos): ADA-positive patients; pred: predictions; SSPB: symmetric signed percentage bias; ζ: median symmetric accuracy.

ζ values represent a measure of accuracy with smaller values indicating higher accuracy, while SSPB values represent a measure of bias with values closer to zero indicating less bias. Figure 2 shows the development of ζ and SSPB values over time for all included population pharmacokinetic models in the ADA negative (Figure 2a,b) and ADA-positive (Figure 2c,d) patient subpopulations. The last category (“all pred”) subsumes unstratified results for all predicted concentrations excluding C_MAP_. The corresponding ζ values were calculated for all 12 models to be within 26–44% (median: 30%) for ADA-negative patients and 77–215% (median: 92%) for ADA-positive patients. SSPB values for all models were within −22–27% (median: 6%) for ADA-negative patients and 8–145% (median: 43%) for ADA-positive patients.

The models exhibiting the highest overall accuracy (lowest ζ) for predicted concentrations in ADA-negative patients were the two models by Fasanmade et al., 2011, both with ζ values of ~26%. Regarding bias in model predictions, four models had absolute SSPB values of ≤5% (with SSPB values for Fasanmade et al., 2009: −3%; Xu et al., 2012: −1%; and the two models from Fasanmade et al., 2011: −5%).

ζ values for predictions in ADA-negative patients increased from a median of 25% (predictions within one month of C_MAP_) and 28% (predictions one to six months after C_MAP_) to 54% (predictions more than six months after C_MAP_) over time (see Figure 2a). In contrast, the median SSPB value for model predictions in ADA-negative patients did not increase over time (median (SSPB _< 1 month_): 7%, median (SSPB _1–6 months_): 8%, median (SSPB _> 6 months_): 2%; Figure 2b). All calculated ζ and SSPB values for each model are listed in Table S1 and Table S2 of the Supplementary Materials.

**Figure 3:**
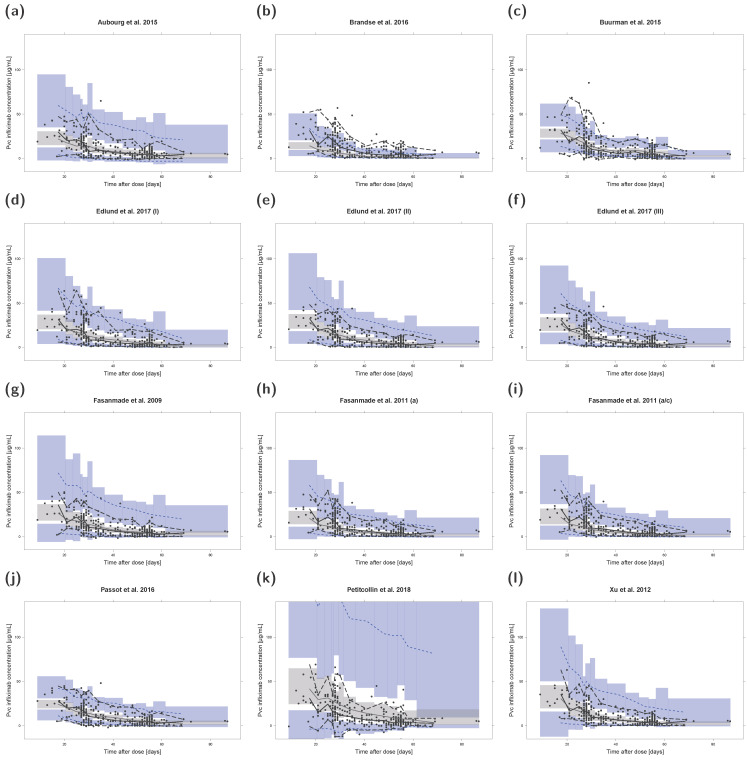
Prediction- and variability-corrected visual predictive checks (pvcVPCs) of serum infliximab concentrations for each investigated population pharmacokinetic model (**a**–**l**). Prediction- and variability-corrected observed concentrations are shown as black circles, observed medians are depicted as black solid lines, 5th and 95th data percentiles as black dashed lines. The model simulations (n = 1000 replicates) are depicted as gray solid lines (median) and blue dashed lines (5th and 95th percentiles). Colored areas represent the simulation-based 95% confidence intervals for the corresponding model-predicted median (gray areas) and 5th and 95th percentiles (blue areas). For ease of comparison, y-axis upper limits were set to 140 µg/mL. Plots with automatic y-axis limits are shown in the Supplementary Materials. a: adults; a/c: adults/children; Pvc: prediction- and variability-corrected.

In ADA-positive patients, predictions of infliximab concentrations were less accurate, especially for concentrations measured within and after one month of first ADA detection (Figure 3c, median (ζ _1st time ADA+ and ≤ 1 month_): 97%, median (ζ _> 1 month ADA+_): 301%). For some models, bias (SSPB) was still low for predictions of concentrations when patients were tested ADA positive for the first time and within one month of detection (Petitcollin et al., 2018: −1%, Fasanmade et al., 2009: −2%, Fasanmade et al., 2011 (adults/children): 5%, Edlund et al., 2017 (II): 9%, and Edlund et al., 2017 (III): −9%) but was high for all models regarding concentrations measured more than one month after patients tested ADA positive for the first time (range of SSPB values: 78–344%).

ζ values for model simulations of C_MAP_ were 0–24% (median: 9%) for ADA-negative patients and 0–43% for ADA-positive patients (median: 12%). The corresponding SSPB values were −24–0% (median: −5%) for ADA-negative patients and −13–22% for ADA-positive patients (median: −4%).

The model by Edlund et al., 2017 (III) included ADA concentrations measured by HMSA (Prometheus Laboratories, San Diego, CA) as a continuous covariate on infliximab CL [43] in contrast to a binary covariate (i.e., ADA negative or ADA positive) as implemented in other evaluated models. However, since model predictions were executed with individual patient covariates imputed from time of C_MAP_, model predictions for later time points could not benefit from continuous measurements of ADA concentrations and other time-varying covariates. In order to examine these potential benefits for the model by Edlund et al., 2017 (III), predictions were also performed with fully informed covariates for the ADA-positive subpopulation and results are depicted in Figure 2c,d (green dashed line). This led to an improvement in both model accuracy and bias, especially for concentrations measured more than one month after patients tested ADA positive for the first time (ζ: 130% vs. 206% and SSPB: 11% vs. 206%).

Predictions with fully informed time-varying covariates were also performed for all other evaluated models for both ADA-negative and ADA-positive patients, and results are shown in Tables S3 and S4, as well as in Figures S1 and S2 in the Supplementary Materials.

### 3.6. Predictions of “Need for Dose Escalation”

According to the American Gastroenterological Association Institute Guideline, the target trough concentration for infliximab is ≥5 µg/mL [13]. In total, 69 serum trough samples from the external data set exhibited infliximab concentrations ≥ 5 µg/mL (no dose escalation needed), 90 trough samples exhibited infliximab concentrations < 5 µg/mL (dose escalation needed). For serum trough samples in which ADA status was negative, 67 samples showed infliximab trough levels ≥5 µg/mL (50%) and 67 showed infliximab trough levels < 5 µg/mL (50%). In contrast, for serum trough samples in which ADA status was positive, only 2 samples showed infliximab levels ≥ 5 µg/mL (8%) and 23 showed infliximab levels < 5 µg/mL (92%).

Table 3 presents the results regarding model abilities to correctly predict the need for dose escalation in the external data set. Results were split into two groups—predictions for serum trough samples in which ADA status was negative and predictions for serum trough samples in which ADA status was positive. Models with the highest accuracy for the ADA-negative sample cohort were the models by Edlund et al., 2017 (II + III), and the models by Fasanmade et al., 2011, with 113/134 (84%) correct predictions. For the ADA-positive sample cohort, the model by Buurman et al., 2015, correctly classified 20 of 25 (80%) concentrations to be above or below the threshold of 5 µg/mL. In summary, the investigated models correctly identified the need for dose escalation (i.e., trough concentration < 5 µg/mL) in 63–89% of cases. In 4–43% of cases a dose escalation would have been recommended (predicted trough concentration < 5 µg/mL) although the measured concentration was above the target concentration.

### 3.7. Prediction- and Variability-Corrected Visual Predictive Checks (pvcVPCs)

The results of pvcVPCs for each investigated population pharmacokinetic model are presented in Figure 3. The pvcVPCs showed a clear overprediction of the 95th percentile of observations for the models by Aubourg et al., 2015, Edlund et al., 2017 (II), Fasanmade et al., 2009, and Xu et al., 2012, but predictions of median infliximab concentrations were reasonable for all four models. The model by Petitcollin et al., 2018, overpredicted and the model by Brandse et al., 2016, underpredicted both the median and 95th percentile of observations. In contrast, the model by Buurman et al., 2015, overpredicted the 5th percentile while slightly underpredicting the 95th percentile. Model simulated median and 95th percentile showed high agreement with the corresponding median/percentile observed for the model by Passot et al., 2016. However, the 5th percentile was overpredicted most of the time. The remaining four models showed high congruence with a slight initial underprediction of the median observations for the two models by Fasanmade et al., 2011, and the model by Edlund et al., 2017 (III).

## 4. Discussion

Several MIPD approaches have recently shown major success in supporting and optimizing dosing regimen selection for various drugs [28,59,60,61,62]. As infliximab trough concentrations exhibit high inter-individual variability and, hence, contribute to a high rate of primary and secondary non-response [9,12,13,14,18] and as infliximab drug exposure is a predictor of clinical response [6,10,11,12], dose selection for infliximab could benefit considerably from population pharmacokinetic modeling and MIPD [31,63]. Consequently, many efforts have been made to analyze infliximab PK, quantifying and explaining inter-individual variability in various population pharmacokinetic models [21,23,24,25,26,27,43,44,45,46,47,48,49,51,52,53,54,55,56,57].

However, for the application of population pharmacokinetic models, an extensive assessment including internal and external evaluation regarding accuracy, robustness, and predictive performance is crucial [64]. While different methods have been applied in the respective internal model evaluations, only a fraction of the models has been evaluated with an independent data set [34,65,66,67,68], and a comprehensive external evaluation for predictive model performances has not been conducted yet. External evaluation with an independent data set allows the evaluation of model performance regarding prediction and variability in patients with a clinical background similar to the internal data set and thus evaluates not only the modeling approach itself, but also other study-related factors [64].

As shown in this analysis, differences in the predictive performances of the 12 investigated models could be observed by external evaluation, and trends in the predictability of infliximab concentrations could be identified for the ADA-negative as well as the ADA-positive subpopulation when using first measured infliximab concentration for estimation of EBEs.

While in ADA-negative patients the absolute SSPB values, as a measure of model bias, did not increase for virtually all models from the predictions of concentrations within one month (SSPB median of 7%) to the predictions of concentrations after more than six months of C_MAP_ (SSPB median of 2%), model accuracy decreased noticeably for predictions of concentrations more than six months after C_MAP_ in this study setting (median (ζ _< 1 month_): 25%, median (ζ _1–6 months_): 28%, median (ζ _> 6 months_): 54%). As this observation also held true for model predictions performed with time-varying covariates, yet to a lesser extent, long-term predictions should be treated carefully because of the deterioration in model accuracy.

Different analytical methods have been used to measure infliximab and ADA concentrations in the population pharmacokinetic analyses, leading to differences in immunogenicity rate [17]. While in some studies, “drug sensitive” methods (ADAs not detectable in the presence of infliximab because of analytic interferences) were used to measure ADA concentrations, “drug-tolerant” assays were applied in other investigations, yielding a much higher rate of ADA-positive patients (up to 62% compared to as low as 1%) [21,51]. Nevertheless, 18 out of 23 models that included ADA patients implemented ADA status as a covariate. The five remaining studies identified only ≤3% of patients as ADA positive or used a risk function of developing ADA. The implementation of ADA status in the majority of models highlights the importance of ADA for the PK of infliximab.

Predictions in ADA-positive patients showed much larger deviations from the corresponding observed values compared to predictions in ADA-negative patients: While prediction accuracy for concentrations before the first ADA-positive blood sample (median(ζ): 31%, range: 17–89%) were similar compared to predictions for ADA-negative patients, predictions became much less accurate as soon as ADA status turned positive (median(ζ): 97%, range 72–361%). As noted, for predictions in this analysis, individual patient covariates for times after C_MAP_ were imputed (last observation carried forward). This especially affected predictions of concentrations in patients showing changes in important covariates such as ADA status. Hence, model predictions were also performed with time-varying covariates (depicted in the Supplementary Materials). While improvements in model predictions were especially noted for concentrations in ADA-positive blood samples, predictions still exhibited ζ values of >100% for concentrations more than one month after patients tested ADA positive for the first time, albeit the inclusion of ADA status in most models.

The study by Edlund et al., 2017, aimed to tackle the challenges of predicting infliximab CL in ADA-positive patients [43]. The corresponding population pharmacokinetic model was based on the models by Fasanmade et al., 2011, and Ternant et al., 2015, with the advancement of including ADA concentrations as a continuous covariate [43]. As a result, when using time-varying covariates, the model by Edlund et al., 2017 (III), showed the highest accuracy and least bias for model predictions in ADA-positive patients for “all pred”. However, the respective model predictions were still less accurate and showed a higher bias compared to predictions for the ADA-negative patient cohort. Additionally, the implemented covariates albumin and IMM in the model by Fasanmade et al., 2011, were not found to be statistically significant with the data set used for model development by Edlund et al., 2017 [43]. This may have contributed to the slightly lower accuracy and higher bias for predictions in ADA-negative patients compared to the models by Fasanmade et al., 2011.

One reason for the observed overprediction in ADA-positive patients could be due to the fact that the exact time of ADA onset is often unknown. ADA-positive patients develop ADA during a time period of unknown length before they test positive for the first time, which is supported by findings from Petitcollin and coworkers [51]. A close and regular monitoring for ADA using drug-tolerant assays as well as the development and application of models identifying predictors of ADA development [69] might help to improve predictive performances for ADA-positive patients.

For predictive performance evaluation in Bayesian forecasting, only the first measured serum infliximab concentration of each patient was used for MAP estimation of EBEs (C_MAP_). Due to the design of the study, C_MAP_ was usually a midpoint concentration and results should be interpreted with this in mind. However, using a midpoint infliximab concentration for estimation of EBEs to predict the subsequent trough level allows potential adjustment of the current dosing interval before infliximab concentrations drop below the target concentration of 5 µg/mL.

As infliximab therapy can be adapted based on trough levels [13], model performances to correctly predict the need for dose escalation (i.e., trough concentration <5 µg/mL) were further investigated. The two examined models by Fasanmade et al., 2011, correctly identified 79 of 90 (88%) trough concentrations to be below the target trough concentration (true positive) while correctly identifying 53 of 69 (77%) trough concentrations to be above the target trough concentration (true negative). This represented the highest classification accuracy of correctly identified infliximab trough samples (132/159, 83%) in this study setting. The model by Brandse et al., 2016 [55], exhibited the highest true positive rate (89%); however, it was accompanied by a low true negative rate of 57%. The model by Passot et al., 2016 [56], showed the highest true negative rate (96%) with a low true positive rate of only 63%. While a high false negative rate yields an increased number of patients with insufficient infliximab levels and, hence, decreased drug effect, a high false positive rate corresponds to an increased number of patients with higher exposure and potentially higher rates of adverse effects (e.g., rate of infection [70]). It should be noted that these measures only reflect whether model predictions were correctly below or above the target trough concentration and do not assess how much predictions deviated from the actual measured concentration.

The investigation of factors leading to differences in model performances was outside the scope of this study, but it is worth mentioning that the vast majority of blood samples from the external data set were collected during infliximab maintenance therapy. In contrast, three examined models were developed with data only from the first 6 weeks (Brandse et al., 2016 [55]), “treatment initiation” (Aubourg et al., 2015 [53]), and the first 22 weeks of infliximab treatment (Passot et al., 2016 [56]), which might have affected prediction performance. The model by Petitcollin et al., 2018, was included in the analysis to study the performance of a model with implemented ADA risk function instead of an ADA covariate, although the model was developed with data from pediatric patients [25]. While the corresponding CL and V_d_ model parameters appear comparable to parameters in infliximab models developed with adult patients [25,43,46,56], the difference in patient cohorts might still explain larger deviations between predicted and measured infliximab concentrations observed in the corresponding pvcVPC and goodness-of-fit plot.

There are some limitations of this analysis, which are discussed in the following paragraphs. Since the study was based on routine therapeutic drug monitoring data, a full dosing schedule was not available for all patients. In such cases, regular dosing from the start of treatment with infliximab and from every change in dosing regimen was assumed. Moreover, sampling times as well as the included patient cohort, treatment period (induction vs. maintenance therapy), analytical methods, or dosing regimens in the external data set could have affected the results of the analysis. Hence, further evaluations with independent data sets should be conducted in future studies.

Another factor influencing the results of an analysis is the choice of quantitative measure. Here, median symmetric accuracy (ζ) and symmetric signed percentage bias (SSPB) values were computed. As illustrated by Morley et al., ζ attenuates the issues with asymmetric penalty and effects of outliers while maintaining interpretability [42]. ζ is a robust measure of accuracy minimizing the effect of the skewness of the distribution of absolute errors [42]. The SSPB estimates the central tendency of the error and can be interpreted similar to a mean percentage error (MPE) [42]. However, in contrast to the MPE, SSPB is not affected by the likely asymmetries in the distribution [42]. As illustrated throughout the analysis, different performance metrics stratified by different patient cohorts (here, ADA-negative and ADA-positive patients) can be of interest when evaluating population pharmacokinetic models in the framework of MIPD. Since different models showed strengths in different measures, we could not appraise which published model was the “best” model, as this also depends on the question of interest and was beyond the scope of this analysis.

Additionally, because of data set incompatibility (e.g., missing covariates) or the lack of reported model implementation details, only 12 of the 25 identified population pharmacokinetic models of infliximab in IBD could be evaluated. While this work already adds a comprehensive analysis to recently published evaluations of single models, an external evaluation including additional covariates (such as the erythrocyte sedimentation rate or fecal calprotectin [21,27]) would be of interest for future studies. Moreover, new modeling approaches (e.g., pharmacokinetic/pharmacodynamic models [27,49]) regarding treatment efficacy could investigate recent findings such as the relation of intestinal microbiota to anti-TNF-α treatment outcome in IBD patients [71,72]. Improvement in gut microbial dysbiosis in IBD patients has been observed during infliximab therapy [71,73], and fecal microbiota has been suggested as a response indicator of infliximab treatment [72]. Future pharmacokinetic/pharmacodynamic studies that examine therapeutic outcome could further investigate this interplay of intestinal microbiota and infliximab therapy. For this, the presented comprehensive external evaluation can also serve as guidance to adopt a suitable population pharmacokinetic model in order to explore these complex response mechanisms.

## 5. Conclusions

This work presents an external evaluation of the predictive performance of 12 published infliximab population pharmacokinetic models in IBD patients using an independent data set. Differences in predictive performance regarding model accuracy, model bias, and need for dose escalation have been observed for both ADA-negative and ADA-positive patients. Using the first measured infliximab concentration for MAP estimation (C_MAP_) in a Bayesian forecasting setting, overall model accuracy decreased for predictions more than six months after C_MAP_ for ADA-negative patients, while bias did not increase. The two investigated models by Fasanmade et al., 2011, showed the highest dose escalation classification accuracy of correctly identified infliximab trough samples (83%) in this study setting. Overall, the investigated population pharmacokinetic models showed a classification accuracy of 75–84% for ADA-negative samples and of 60–80% for ADA-positive samples. The results of this predictive performance evaluation could help to guide and plan future MIPD approaches with infliximab population pharmacokinetic models to improve individual dosing strategies and prevent infliximab trough concentrations dropping below the target concentration. Yet clinical application needs to be tested and confirmed in larger, prospective clinical trials. In comparison to predictions for ADA-negative patients, model predictions of serum concentrations for ADA-positive patients showed lower accuracy and higher bias. Thus, predictions with population pharmacokinetic models remain particularly challenging for ADA-positive patients and for patients with unknown ADA status.

## Figures and Tables

**Table 2:** Clinical and demographic patient characteristics.

Characteristic	Median or No.	Range	IQR
Patients, n	105		
Sex, female, n (%)	50 (48)		
Patients with CD, n (%)	76 (72)		
Patients with UC, n (%)	29 (28)		
ADA-positive patient status, n (%)	22 (21)		
IMM ^1^, n (%)	17 (16)		
Nonsmoker, n (%)	35 (33)		
Smoker, n (%)	41 (39)		
Past smoker, n (%)	28 (27)		
Unknown smoking status, n (%)	1 (1)		
Body weight ^1^ [kg]	70	47–115	59–80
Height ^1^ [cm]	171	155–190	165–178
Albumin ^1^ [g/dL]	4.35	2.53–5.08	4.12–4.54
CRP ^1^ [mg/dL]	0.29	0.02–7.49	0.11–0.49
HBI ^1^	1	0–18	1–3
Total serum samples, n	336		
ADA-positive serum samples, n (%)	49 (15)		

Note: ADA: anti-drug antibodies; CD: Crohn’s disease; CRP: C-reactive protein; HBI: Harvey–Bradshaw index; IMM: immunomodulators (including azathioprine and methotrexate); IQR: interquartile range; No.: number; UC: ulcerative colitis; ^1^ at the time of first drug sampling.

**Table 3:** Predictions of “need for dose escalation” (i.e., trough concentration <5 µg/mL [13]).

	ADA Negative					ADA Positive				
Dose Escalation Needed? (Cobs < 5 µg/mL)	Yes (n = 67)		No (n = 67)			Yes (n = 23)		No (n = 2)		
Correctly Predicted?	Yes	No	Yes	No	Accuracy	Yes	No	Yes	No	Accuracy
Aubourg et al., 2015	48	19	63	4	82.8%	13	10	2	0	60.0%
Brandse et al., 2016	62	5	39	28	75.4%	18	5	0	2	72.0%
Buurman et al., 2015	38	29	62	5	74.6%	19	4	1	1	80.0%
Edlund et al., 2017 (I)	51	16	61	6	83.6%	16	7	2	0	72.0%
Edlund et al., 2017 (II)	50	17	63	4	84.3%	15	8	1	1	64.0%
Edlund et al., 2017 (III)	50	17	63	4	84.3%	16	7	1	1	68.0%
Fasanmade et al., 2009	54	13	58	9	83.6%	17	6	1	1	72.0%
Fasanmade et al., 2011 (a/c)	60	7	53	14	84.3%	19	4	0	2	76.0%
Fasanmade et al., 2011 (a)	60	7	53	14	84.3%	19	4	0	2	76.0%
Passot et al., 2016	44	23	64	3	80.6%	13	10	2	0	60.0%
Petitcollin et al., 2018	62	5	48	19	82.1%	15	8	0	2	60.0%
Xu et al., 2012	56	11	52	15	80.6%	18	5	1	1	76.0%

Note: a: adults; a/c: adults/children; ADA: anti-drug antibody; C_obs_: observed trough concentration.

## Data Availability

Not applicable.

## Supplementary Materials

The following are available online at https://www.mdpi.com/article/10.3390/pharmaceutics13091368/s1, Electronic Supplementary Materials: Additional evaluation results. Figure S1: Individual predicted versus observed serum infliximab concentrations for the population pharmacokinetic model by Petitcollin et al. 2018. Concentrations of anti-drug antibody (ADA) negative patients are shown in turquoise, concentrations of ADA positive patients in pink. Concentrations used for maximum a posteriori (MAP) estimation (CMAP) are depicted as triangles, the remaining symbols depict predictions in different time intervals after CMAP. The black solid line represents the line of identity, grey dashed lines mark the target trough concentration of 5 µg/mL. (neg): ADA negative patients; (pos): ADA positive patients, Figure S2: Model prediction accuracy (ζ, a and b) and bias (SSPB, c and d) for anti-drug antibody (ADA) negative pa-tients over time. The left panel shows ζ and SSPB values for model predictions with fixed covariates determined at the time of the first measured serum infliximab concentration of each patient (CMAP), the right panel shows ζ and SSPB val-ues for model predictions with time-varying covariates. “all pred” covers all predicted concentrations excluding CMAP. Numbers in parentheses refer to the number of observed concentrations in the respective time interval. (neg): ADA negative patients, pred: predictions; SSPB: symmetric signed percentage bias; ζ: median symmetric accuracy, Figure S3: Model prediction accuracy (ζ, a and 2b) and bias (SSPB, c and d) for anti-drug antibody (ADA) positive pa-tients over time. The left panel shows ζ and SSPB values for model predictions with fixed covariates determined at the time of the first measured serum infliximab concentration of each patient (CMAP), the right panel shows ζ and SSPB val-ues for model predictions with time-varying covariates. “all pred” covers all predicted concentrations excluding CMAP. Numbers in parentheses refer to the number of observed concentrations in the respective time interval. (pos): ADA pos-itive patients, pred: predictions; SSPB: symmetric signed percentage bias; ζ: median symmetric accuracy, Figure S4: Prediction- and variability-corrected visual predictive check (pvcVPC) of serum infliximab concentrations for the population pharmacokinetic model by Petitcollin et al. 2018. Prediction- and variability-corrected observed concen-trations are shown as black circles, observed median is depicted as black solid line, 5th and 95th data percentiles as black dashed lines. The model simulations (n = 1000 replicates) are depicted as grey solid line (median) and blue dashed lines (5th and 95th percentiles). Colored areas represent the simulation-based 95% confidence intervals for the corresponding model-predicted median (grey areas) and 5th and 95th percentiles (blue areas). Pvc: prediction- and variability-corrected, Table S1: ζ and SSPB values for model predictions with fixed covariates at time of CMAP for ADA negative patients, Table S2: ζ and SSPB values for model predictions with fixed covariates at time of CMAP for ADA positive patients, Table S3: ζ and SSPB values for model predictions with time-varying covariates for ADA negative patients, Table S4: ζ and SSPB values for model predictions with time-varying covariates for ADA positive patients.
