# Supplementary material for: External Model Performance Evaluation of Twelve Infliximab Population Pharmacokinetic Models in Patients with Inflammatory Bowel Disease
Source: Pharmaceutics. 2021 Aug 31;13(9):1368. doi: 10.3390/pharmaceutics13091368 (PMC8468301; doi:10.3390/pharmaceutics13091368)
Supplement: Supplementary file 1 [file pharmaceutics-13-01368-s001.zip › pharmaceutics-1343942-supplementary.pdf]

pharmaceutics

Supplementary Material: External Model Performance Evaluation of Twelve Infliximab Population Pharmacokinetic Models in Patients with Inflammatory Bowel Disease

Christina Schräpel, Lukas Kovar, Dominik Selzer, Ute Hofmann, Florian Tran, Walter Reinisch, Matthias Schwab and Thorsten Lehr

1. Assumptions for model implementations of investigated population pharmacokinetic models

In the publication by Brandse et al. 2016, the additive residual error of 0.26 was reported without a unit [1]. The unit μg/mL, which was used throughout the respective publication to report IFX concentrations was assumed.

In the publication by Buurman et al. 2015 [2], the equation for calculating the central volume of distribution was presented as

$$V_i = V_{pop} * 0.964 * (HBI - 6) \quad \text{(S1)}$$

with HBI being the Harvey-Bradshaw index and Vpop being the central volume of distribution of the typical patient. Using this equation, Vi would become negative for HBI values < 6. The respective publication reported an HBI range of the internal dataset of 3–24. With the help of information from the text ("For V, a significant and clinically relevant effect was found for the HBI at baseline, a higher value resulting in lower values of V" [2]) and from Table 3 (θHBI is −3.6 per HBI point) in the respective publication [2], the equation was changed to:

$$V_i = V_{pop} \times (1 - 0.036 \times (HBI - 6)) \quad \text{(S2)}$$

indicating a lower central volume of distribution with higher HBI values.

In the publication by Aubourg et al. 2015, contradictory units were displayed for the clearance (CL) and intercompartmental clearance (Q) parameters (L/h and L/day, respectively) when comparing values from text and Table 1 [3]. However, in Table 1, also CL and Q parameters from other infliximab population pharmacokinetic models were displayed. By comparing these parameters to the respective publications, it becomes clear, that the correct unit is L/h which was used for our model implementation.

For predictions with the model by Edlund et al. 2017 (III), ADA concentrations reported below the lower limit of quantification were treated as zero as suggested in the respective publication [4].

The full-text version of the publication by Xu et al. 2012 [5] could not be identified. As a result, the information for model implementation were gathered from the abstract as well as from the publication by Wojciechowski and coworkers [6], who used the model by Xu et al. 2012 to simulate individual pharmacokinetic parameters for a virtual study population and who reported the modeling information of the model by Xu et al. 2012.

For model predictions with time-varying covariates, changes in covariates over time were acknowledged (e.g. change in ADA status, HBI, weight) and used for model computations. Missing continuous covariates were imputed by median values and missing categorical covariates by the mode (most frequent value).

2 Predictive model performance evaluation

2.1. Goodness-of-fit plots

The goodness-of-fit plot showing the individual predicted versus observed serum infliximab concentrations for the population pharmacokinetic model by Petitcollin et al. 2018 [7] including the concentration which was cut-off in the main manuscript is depicted in Figure S1.

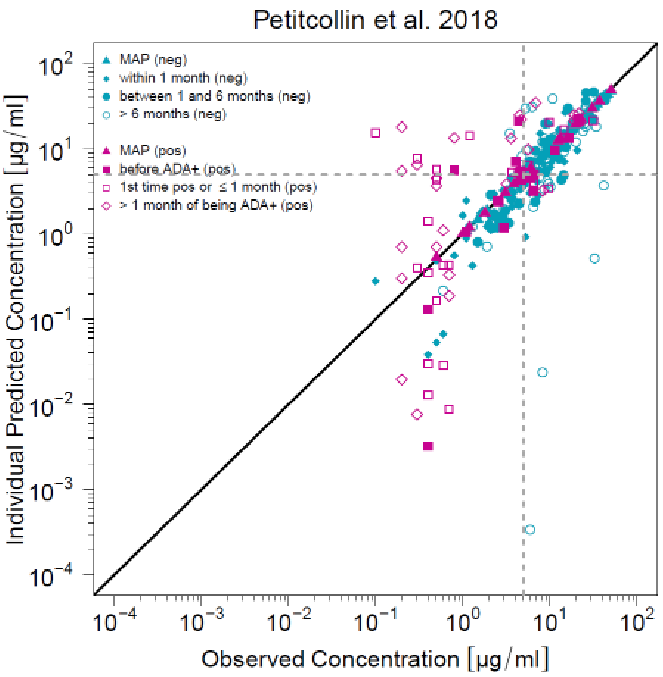

Figure S1. Individual predicted versus observed serum infliximab concentrations for the population pharmacokinetic model by Petitcollin et al. 2018. Concentrations of anti-drug antibody (ADA) negative patients are shown in turquoise, concentrations of ADA positive patients in pink. Concentrations used for maximum a posteriori (MAP) estimation (C_{MAP}) are depicted as triangles, the remaining symbols depict predictions in different time intervals after C_{MAP}. The black solid line represents the line of identity, grey dashed lines mark the target trough concentration of 5 µg/mL. (neg): ADA negative patients; (pos): ADA positive patients.

2.2 Accuracy and bias of model predictions

The calculated median symmetric accuracy (ζ) and symmetric signed percentage bias (SSPB) values for all included population pharmacokinetic models are shown in Tables S1 to S4. Tables S1 and S2 list the results for model predictions with fixed covariates determined at the time of the first measured serum infliximab concentration of each patient (CMAP), while Tables S3 and S4 list the results for model predictions with time-varying covariates. Tables S1 and S3 provide the calculated ζ and SSPB values for the ADA negative patient cohort. Tables S2 and S4 provide the ζ and SSPB values for the ADA positive patient cohort. In addition, Figures S2 and S3 show the corresponding visual depiction of ζ and SSPB values.

Table S1. ζ and SSPB values for model predictions with fixed covariates at time of C_{MAP} for ADA negative patients.

Model	ζ (%)					SSPB (%)				
	MAP	< 1 m	1–6 m	> 6 m	all pred	MAP	< 1 m	1–6 m	> 6 m	all pred
Aubourg et al. 2015	9.2	20.8	32.1	54.7	28.0	−8.3	17.3	19.0	5.0	15.6
Brandse et al. 2016	23.7	33.7	28.9	49.3	33.2	−23.7	−27.8	−21.9	−18.0	−22.0
Buurman et al. 2015	18.8	43.8	35.0	65.2	43.5	−1.6	40.4	15.3	21.2	27.5
Edlund et al. 2017 (I)	6.2	26.5	28.0	53.8	33.1	−1.5	11.5	17.6	11.0	13.4
Edlund et al. 2017 (II)	5.1	24.6	27.3	53.5	30.5	−1.9	12.0	17.0	9.2	12.0
Edlund et al. 2017 (III)	4.5	23.2	27.2	54.5	29.1	−2.4	11.8	16.1	8.5	12.5
Fasanmade et al. 2009	16.1	24.9	23.5	50.6	27.3	−14.1	−3.5	0.4	−6.1	−2.6
Fasanmade et al. 2011 (a)	14.7	24.7	21.0	54.9	26.4	−14.4	−8.6	1.0	−2.2	−5.3
Fasanmade et al. 2011 (a/c)	13.3	23.6	21.5	53.0	25.5	−12.9	−8.5	0.3	−1.2	−5.2
Passot et al. 2016	0.3	33.1	46.4	49.3	37.7	−0.2	26.9	41.0	20.2	26.6
Petitcollin et al. 2018	2.5	30.0	34.9	69.3	39.5	−2.5	−20.1	−8.6	−37.3	−19.8
Xu et al. 2012	18.0	25.6	20.4	57.5	27.1	−16.9	1.6	−3.2	−6.5	−0.6

a: adults; a/c: adults/children; ADA: anti-drug antibody; m: month; pred: predicted; SSPB: symmetric signed percentage bias; ζ: median symmetric accuracy. Abbreviations for time intervals refer to descriptions in the main manuscript.

Table S2. ζ and SSPB values for model predictions with fixed covariates at time of C_{MAP} for ADA positive patients.

Model	ζ [%]					SSPB [%]				
	MAP	before ADA+	1st time ADA+ or ≤ 1 m	> 1 m after ADA+	all pred	MAP	before ADA+	1st time ADA+ or ≤ 1 m	> 1 m after ADA+	all pred
Aubourg et al. 2015	10.7	33.9	98.2	301.9	92.8	-5.2	32.8	82.6	301.9	78.7
Brandse et al. 2016	18.4	51.5	278.6	384.4	214.9	-12.8	-30.0	-66.2	180.7	8.1
Buurman et al. 2015	43.1	88.9	361.4	175.1	144.8	22.3	70.0	361.4	175.1	144.8
Edlund et al. 2017 (I)	10.6	41.3	72.1	300.4	86.4	-9.5	-7.5	30.1	300.4	51.1
Edlund et al. 2017 (II)	6.8	23.9	77.3	344.3	85.4	0.4	7.8	9.4	344.3	37.6
Edlund et al. 2017 (III)	5.2	24.9	91.2	205.5	90.8	-0.3	6.3	-9.4	205.5	25.9
Fasanmade et al. 2009	15.1	31.9	79.9	330.1	111.7	-2.9	-4.3	-2.0	330.1	48.4
Fasanmade et al. 2011 (a)	15.8	28.5	107.5	250.9	85.1	-9.9	-25.6	13.5	191.6	14.1
Fasanmade et al. 2011 (a/c)	14.1	29.9	95.4	254.1	83.6	-9.2	-13.8	5.5	200.6	15.6
Passot et al. 2016	0.3	17.1	144.5	347.7	128.0	0.1	14.5	78.4	330.1	73.0
Petitcollin et al. 2018	2.3	29.4	106.3	269.9	108.5	-2.0	-10.7	-0.5	77.6	16.4
Xu et al. 2012	16.5	38.7	80.7	303.3	77.1	-8.3	3.5	54.3	303.3	50.1

ζ: median symmetric accuracy; a: adults; a/c: adults/children; ADA: anti-drug antibody, ADA+: anti-drug antibody positive; m: month; pred: predicted; SSPB: symmetric signed percentage bias. Abbreviations for time intervals refer to descriptions in the main manuscript.

Table S3. ζ and SSPB values for model predictions with time-varying covariates for ADA negative patients.

Model	ζ [%]					SSPB [%]				
	MAP	< 1 m	1 - 6 m	> 6 m	all pred	MAP	< 1 m	1 - 6 m	> 6 m	all pred
Aubourg et al. 2015	9.2	20.9	32.2	54.7	27.8	-8.3	17.3	19.1	5.7	15.6
Brandse et al. 2016	23.7	33.5	30.5	45.9	33.1	-23.7	-27.9	-26.3	-21.6	-22.6
Buurman et al. 2015	18.8	44.9	31.0	55.8	44.3	-1.6	43.4	15.2	19.3	26.7
Edlund et al. 2017 (I)	6.2	27.3	26.5	54.2	31.0	-1.5	11.5	16.0	10.5	12.9
Edlund et al. 2017 (II)	5.1	24.6	27.0	52.9	30.2	-1.9	12.0	14.4	10.3	11.9
Edlund et al. 2017 (III)	4.5	23.2	25.3	52.5	28.6	-2.4	11.8	10.9	-1.8	10.4
Fasanmade et al. 2009	16.1	25.1	24.1	47.7	26.0	-14.1	-3.6	0.6	-3.3	-2.5
Fasanmade et al. 2011 (a)	14.7	24.5	16.4	55.0	25.5	-14.4	-8.3	-2.5	-1.2	-5.1
Fasanmade et al. 2011 (a/c)	13.3	23.4	20.1	52.9	24.9	-12.9	-8.8	-1.4	0.2	-5.5
Passot et al. 2016	0.3	33.1	46.4	49.8	37.5	-0.2	27.1	41.0	16.6	26.4
Petitcollin et al. 2018	2.5	30.3	36.3	79.9	42.0	-2.5	-20.7	-16.5	-48.2	-22.0
Xu et al. 2012	18.0	25.2	16.6	57.3	26.8	-16.9	1.3	-5.4	-6.2	-3.9

ζ: median symmetric accuracy; a: adults; a/c: adults/children; ADA: anti-drug antibody; m: month; pred: predicted; SSPB: symmetric signed percentage bias. Abbreviations for time intervals refer to descriptions in the main manuscript.

Table S4. ζ and SSPB values for model predictions with time-varying covariates for ADA positive patients.

**Model**	**ζ [%]**					**SSPB [%]**				
	MAP	before ADA+	1st time ADA+ or ≤ 1 m	> 1 m after ADA+	all pred	MAP	before ADA+	1st time ADA+ or ≤ 1 m	> 1 m after ADA+	all pred
Aubourg et al. 2015	10.7	33.9	98.2	305.8	92.8	-5.2	32.8	85.2	305.8	79.7
Brandse et al. 2016	18.4	52.5	288.9	231.2	156.7	-12.8	-21.0	-113.3	-141.0	-56.5
Buurman et al. 2015	43.1	88.9	156.3	137.9	130.6	22.3	70.0	128.7	103.1	103.6
Edlund et al. 2017 (I)	10.6	40.6	72.1	309.9	82.2	-9.5	-7.5	30.1	309.9	51.7
Edlund et al. 2017 (II)	6.8	23.9	114.6	156.0	86.5	0.4	7.8	9.4	142.9	22.4
Edlund et al. 2017 (III)	5.2	24.9	109.8	129.5	71.2	-0.3	6.3	-14.4	11.4	6.4
Fasanmade et al. 2009	15.1	28.9	197.0	188.7	93.9	-2.9	-3.8	-33.3	153.8	14.3
Fasanmade et al. 2011 (a)	15.8	30.0	114.2	125.6	77.0	-9.9	-15.3	-18.7	77.0	-1.1
Fasanmade et al. 2011 (a/c)	14.1	31.0	95.4	132.3	77.8	-9.2	-13.8	-11.2	79.0	-1.9
Passot et al. 2016	0.3	17.1	144.5	354.8	127.8	0.1	14.5	78.4	340.0	66.9
Petitcollin et al. 2018	2.3	75.3	214.4	399.5	183.1	-2.0	-18.6	12.3	43.1	1.8
Xu et al. 2012	16.5	38.1	98.8	161.0	86.7	-8.3	2.9	51.6	134.9	58.8

ζ: median symmetric accuracy; a: adults; a/c: adults/children; ADA: anti-drug antibody, ADA+: anti-drug antibody positive; m: month; pred: predicted; SSPB: symmetric signed percentage bias. Abbreviations for time intervals refer to descriptions in the main manuscript.

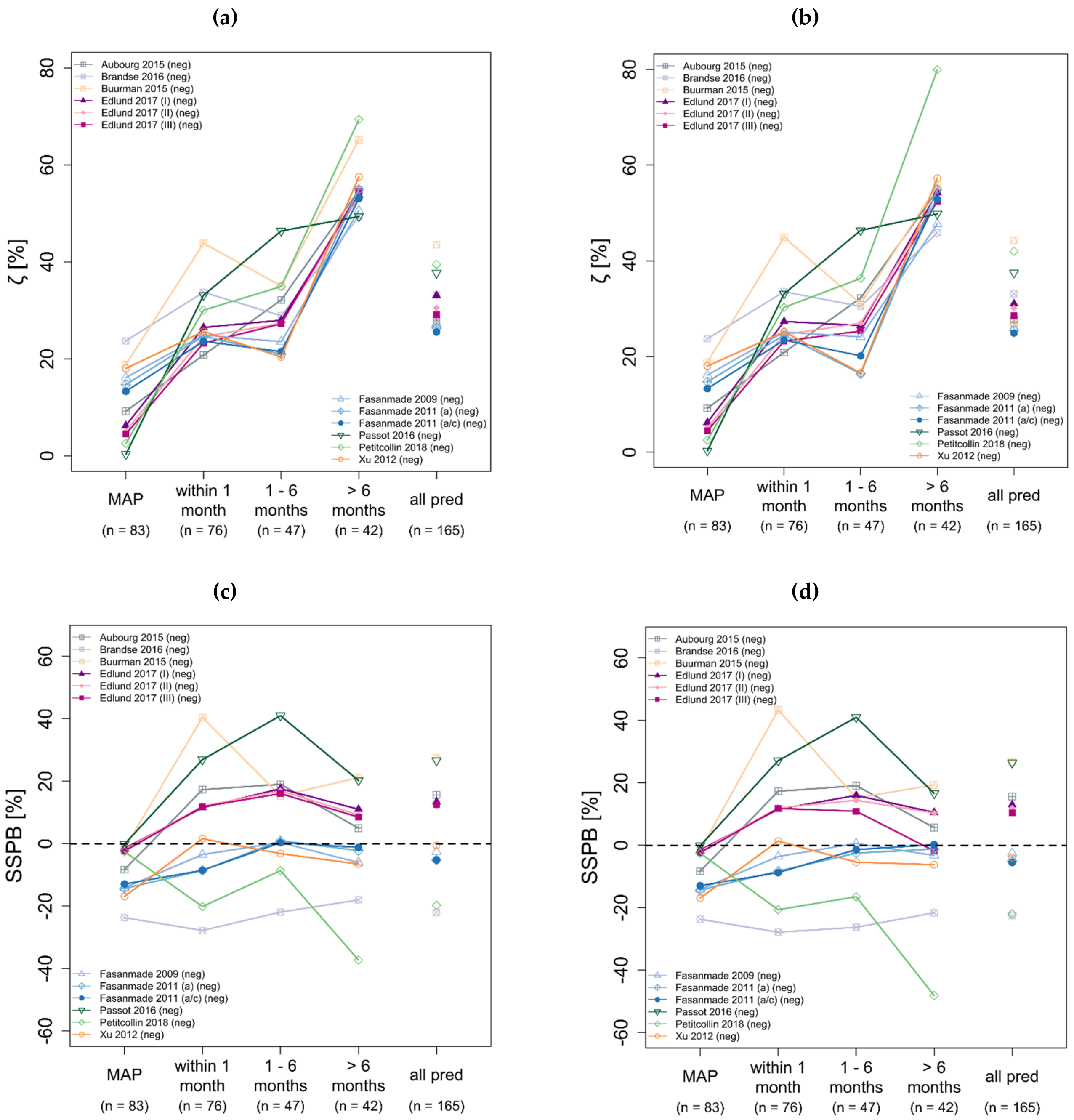

Figure S2. Model prediction accuracy (ζ, **a** and **b**) and bias (SSPB, **c** and **d**) for anti-drug antibody (ADA) negative patients over time. The left panel shows ζ and SSPB values for model predictions with fixed covariates determined at the time of the first measured serum infliximab concentration of each patient (C_{MAP}), the right panel shows ζ and SSPB values for model predictions with time-varying covariates. "all pred" covers all predicted concentrations excluding C_{MAP}. Numbers in parentheses refer to the number of observed concentrations in the respective time interval. (neg): ADA negative patients, pred: predictions; SSPB: symmetric signed percentage bias; ζ: median symmetric accuracy.

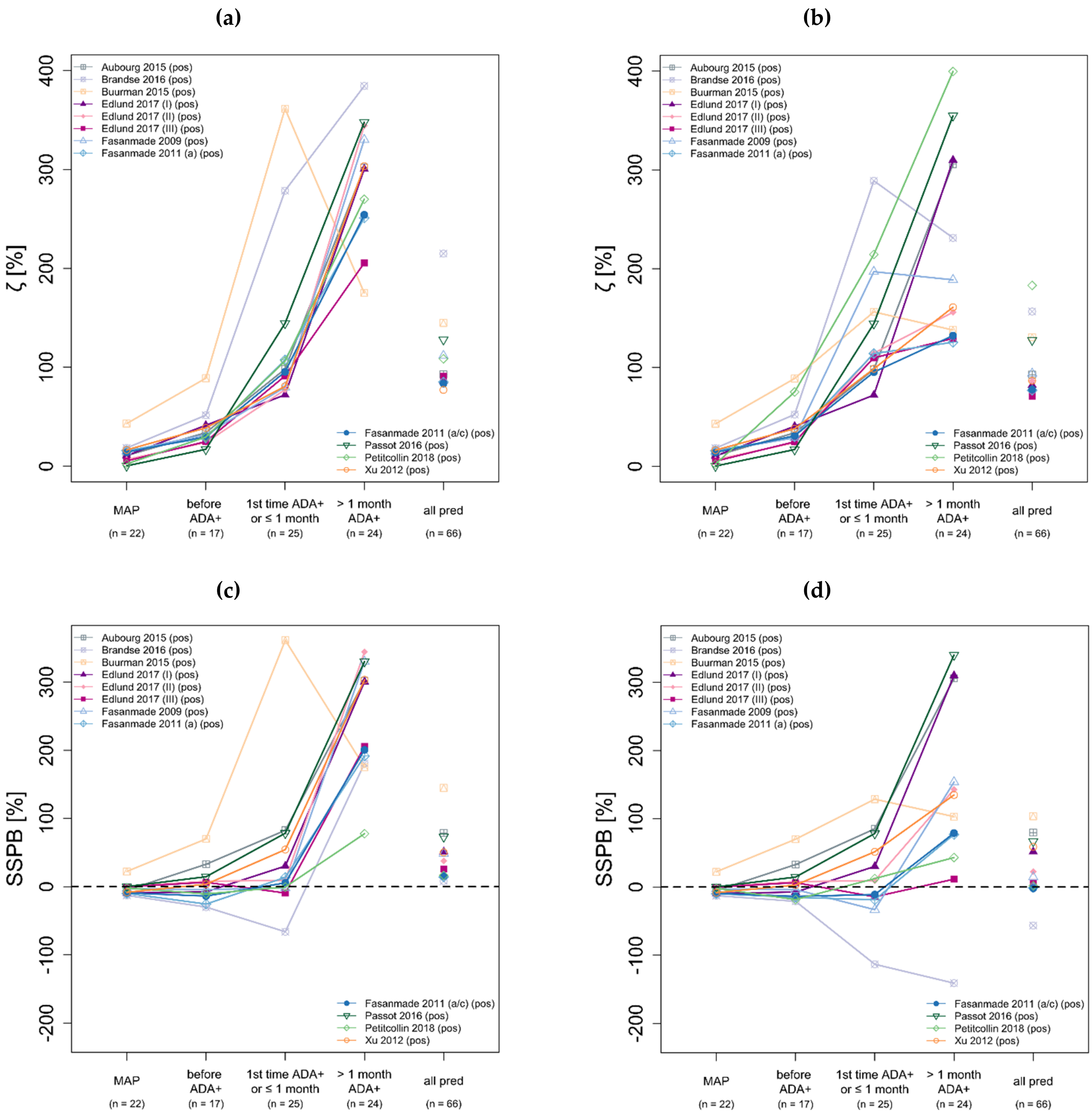

Figure S3. Model prediction accuracy (ζ, **a** and **b**) and bias (SSPB, **c** and **d**) for anti-drug antibody (ADA) positive patients over time. The left panel shows ζ and SSPB values for model predictions with fixed covariates determined at the time of the first measured serum infliximab concentration of each patient (C_{MAP}), the right panel shows ζ and SSPB values for model predictions with time-varying covariates. "all pred" covers all predicted concentrations excluding C_{MAP}. Numbers in parentheses refer to the number of observed concentrations in the respective time interval. (pos): ADA positive patients, pred: predictions; SSPB: symmetric signed percentage bias; ζ: median symmetric accuracy.

2.3 Prediction- and variability-corrected visual predictive checks (pvcVPCs)

In this section, the pvcVPC for the population pharmacokinetic model by Petitcollin et al. 2018 is shown with automatic (full range) y-axis limits.

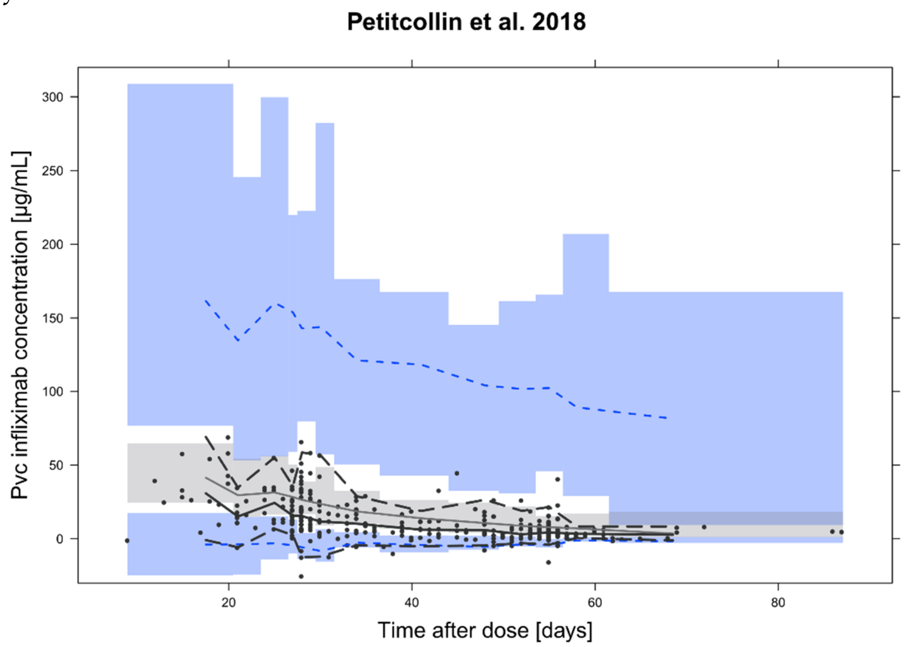

Figure S4. Prediction- and variability-corrected visual predictive check (pvcVPC) of serum infliximab concentrations for the population pharmacokinetic model by Petitcollin et al. 2018. Prediction- and variability-corrected observed concentrations are shown as black circles, observed median is depicted as black solid line, 5th and 95th data percentiles as black dashed lines. The model simulations (n=1000 replicates) are depicted as grey solid line (median) and blue dashed lines (5th and 95th percentiles). Colored areas represent the simulation-based 95% confidence intervals for the corresponding model-predicted median (grey areas) and 5th and 95th percentiles (blue areas). Pvc: prediction- and variability-corrected.

References

1. Brandse, J.F.; Mathôt, R.A.; van der Kleij, D.; Rispens, T.; Ashruf, Y.; Jansen, J.M.; Rietdijk, S.; Löwenberg, M.; Ponsioen, C.Y.; Singh, S.; et al. Pharmacokinetic Features and Presence of Antidrug Antibodies Associate With Response to Infliximab Induction Therapy in Patients With Moderate to Severe Ulcerative Colitis. *Clin. Gastroenterol. Hepatol.* **2016**, *14*, 251-258.e2, doi:10.1016/j.cgh.2015.10.029.
2. Buurman, D.J.; Maurer, J.M.; Keizer, R.J.; Kosterink, J.G.W.; Dijkstra, G. Population pharmacokinetics of infliximab in patients with inflammatory bowel disease: Potential implications for dosing in clinical practice. *Aliment. Pharmacol. Ther.* **2015**, *42*, 529–539, doi:10.1111/apt.13299.
3. Aubourg, A.; Picon, L.; Lecomte, T.; Bejan-Angoulvant, T.; Paintaud, G.; Ternant, D. A robust estimation of infliximab pharmacokinetic parameters in Crohn's disease. *Eur. J. Clin. Pharmacol.* **2015**, *71*, 1541–1542, doi:10.1007/s00228-015-1942-8.
4. Edlund, H.; Steenholdt, C.; Ainsworth, M.A.; Goebgen, E.; Brynskov, J.; Thomsen, O.; Huisinga, W.; Kloft, C. Magnitude of Increased Infliximab Clearance Imposed by Anti-infliximab Antibodies in Crohn's Disease Is Determined by Their Concentration. *AAPS J.* **2017**, *19*, 223–233, doi:10.1208/s12248-016-9989-8.
5. Xu, Z.; Mould, D.; Hu, C.; Al., E. Population pharmacokinetic analysis of infliximab in pediatrics using integrated data from six clinical trials. *Clin Pharmacol Drug Dev.* **2012**, *1*, 203.
6. Wojciechowski, J.; Upton, R.N.; Mould, D.R.; Wiese, M.D.; Foster, D.J.R. Infliximab Maintenance Dosing in Inflammatory Bowel Disease: an Example for In Silico Assessment of Adaptive Dosing Strategies. *AAPS J.* **2017**, *19*, 1136–1147, doi:10.1208/s12248-017-0082-8.
7. Petitcollin, A.; Leuret, O.; Tron, C.; Lemaitre, F.; Verdier, M.C.; Paintaud, G.; Bouguen, G.; Willot, S.; Bellissant, E.; Ternant, D. Modeling Immunization to Infliximab in Children with Crohn's Disease Using Population Pharmacokinetics: A Pilot Study. *Inflamm. Bowel Dis.* **2018**, *24*, 1745–1754, doi:10.1093/ibd/izy129.
